# Limits of Executive Control: Sequential Effects in Predictable Environments

**DOI:** 10.1177/0956797616631990

**Published:** 2016-03-21

**Authors:** Frederick Verbruggen, Amy McAndrew, Gabrielle Weidemann, Tobias Stevens, Ian P. L. McLaren

**Affiliations:** 1 School of Psychology, University of Exeter; 2 School of Social Sciences and Psychology, Western Sydney University

**Keywords:** executive control, expectancy, sequential effects, motor-evoked potentials, transcranial magnetic stimulation, open data

## Abstract

Cognitive-control theories attribute action control to executive processes that modulate behavior on the basis of expectancy or task rules. In the current study, we examined corticospinal excitability and behavioral performance in a go/no-go task. Go and no-go trials were presented in runs of five, and go and no-go runs alternated predictably. At the beginning of each trial, subjects indicated whether they expected a go trial or a no-go trial. Analyses revealed that subjects immediately adjusted their expectancy ratings when a new run started. However, motor excitability was primarily associated with the properties of the previous trial, rather than the predicted properties of the current trial. We also observed a large latency cost at the beginning of a go run (i.e., reaction times were longer for the first trial in a go run than for the second trial). These findings indicate that actions in predictable environments are substantially influenced by previous events, even if this influence conflicts with conscious expectancies about upcoming events.

Scientists often attribute goal-directed behavior to a top-down executive-control system (Verbruggen, McLaren, & Chambers, 2014). One of its main functions is biasing competition between stimulus or response options on the basis of expectancy or task rules. For example, when the control system predicts a certain action, it can preactivate the motor network, biasing action selection and reducing the response latency of the anticipated action (Bestmann, 2012). However, in environments in which there is much uncertainty about upcoming events, decision making also relies on choice history and other subtle sources of evidence that are not consciously monitored (Bode et al., 2014). Consequently, response latencies and expectancy can be dissociated under conditions of uncertainty (Perruchet, Cleeremans, & Destrebecqz, 2006). Thus, action control may depend on an interplay between top-down and bottom-up factors.

Top-down biasing is assumed to reduce the influence of bottom-up factors. Nevertheless, task inertia and sequential effects have been observed in many executive-control paradigms (e.g., Egner, 2014), even when subjects are given the opportunity to bias activation in advance (e.g., Vandierendonck, Liefooghe, & Verbruggen, 2010). Top-down control may have been suboptimal in previous studies because there was usually some remaining uncertainty about upcoming events (i.e., the environment was never entirely predictable). Alternatively, the bottom-up influences could indicate that there are limits to executive control. Therefore, in the present study, we explored whether action and expectancy could be dissociated in entirely predictable environments. Finding such a dissociation would indicate that, even when people can precisely predict what will happen on the next trial, bottom-up factors still influence performance; in other words, people would always be affected by what happened in the (recent) past.

We examined top-down control in a go/no-go task in which runs of go and no-go trials alternated predictably. On each trial, we obtained expectancy ratings and measured corticospinal motor excitability before the presentation of the go or no-go stimulus. We also measured response latencies on go trials. We predicted, on the basis of the top-down biasing account, that motor excitability would increase and that response latencies would decrease when subjects expected a go response (indicating preactivation of the motor network, as noted earlier); by contrast, excitability should decrease and latencies should increase when subjects expected a no-go response (see, e.g., Bestmann, 2012; Leocani, Cohen, Wassermann, Ikoma, & Hallett, 2000; Verbruggen & Logan, 2009).

## Method

Subjects performed a predictable go/no-go task. On both go and no-go trials, we asked subjects to indicate whether they expected a go or no-go trial, and we measured muscular responses (motor evoked potentials, or MEPs) to transcranial magnetic stimulation (TMS) of the primary motor cortex. MEPs are a measure of corticospinal excitability (Hallett, 2007) and are very useful for examining anticipatory influences on the premotor cortex and motor cortex because they can be measured before a stimulus appears. On go trials, we also examined response latencies, because differences in latency between go trials can also reveal response biases.

### Subjects

Sixteen students (14 women; mean age = 20.9 years, age range = 18–24 years) from the University of Exeter participated in this experiment. All subjects were right-handed and were paid £15 for their participation. The experiment was approved by the local research ethics committee at the School of Psychology, University of Exeter. Written informed consent was obtained after the nature and possible consequences of the study were explained. One subject was replaced because an interview at the end of the experiment indicated that he or she did not realize that trials were presented in runs of five, even though this was explicitly mentioned during the instruction phase. Sample size was determined in advance, assuming a large effect of the entirely predictable run types (i.e., go and no-go).

### Behavioral task

**Fig. 1.**
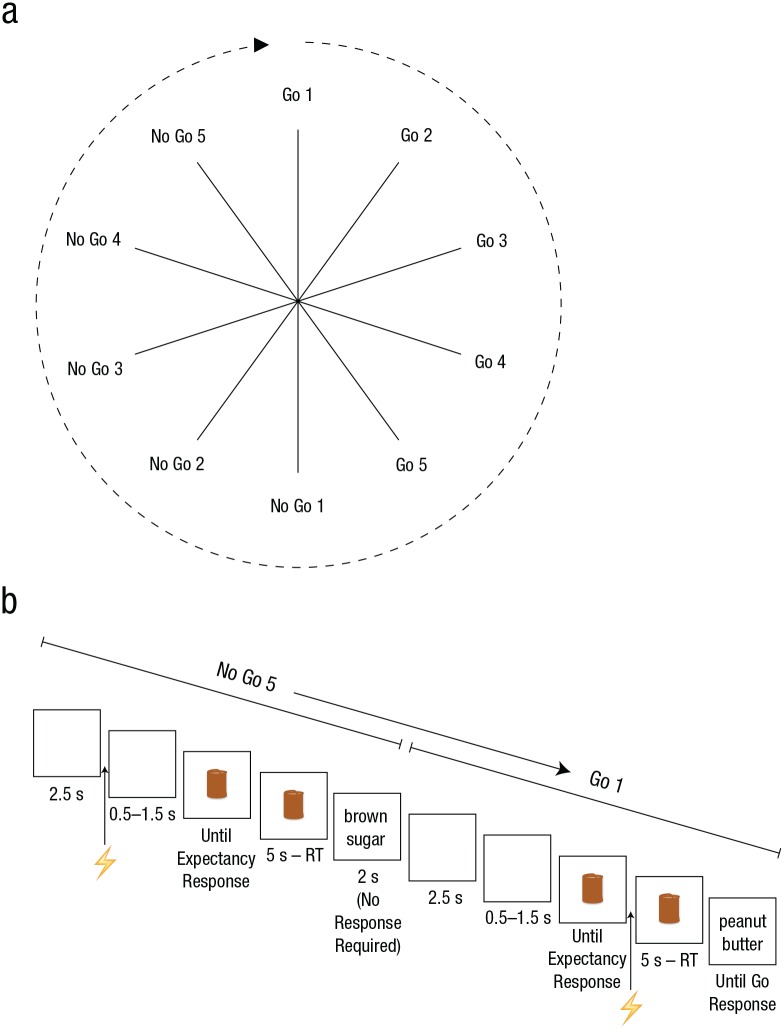
The run sequence (a) and an example of the trial sequence (b). Go and no-go trials were presented in runs of five. The predictable alternation of go and no-go runs produced the cycle of events shown; each spoke represents a trial (a). Each trial started with the presentation of a blank screen. After a variable time interval (3–4 s), a brown cylinder appeared, and subjects rated the extent to which they thought the no-go stimulus would appear. The cylinder remained on screen for 5 s. Then, the go stimulus (e.g., “peanut butter”) or no-go stimulus (e.g., “brown sugar”) appeared. A go stimulus remained on-screen until a response was made, whereas the no-go stimulus disappeared after 2 s. A transcranial-magnetic-stimulation pulse (indicated by the lightning bolt) was delivered at one of two different time points in a trial, either 2.5 s into the blank interval (illustrated here in the no-go trial) or immediately after the subject had provided an expectancy rating (shown here in the go trial). RT = reaction time. See the Method section for further details.

The experiment was run on an iMac with a 21.5-in. screen using Psychophysics Toolbox (Version 3.0.10; Brainard, 1997). After the motor threshold was determined (see Transcranial Magnetic Stimulation section), task instructions were presented on the screen and confirmed verbally with the subjects. Subjects were told that go and no-go trials would be presented in runs of five (Fig. 1a), and that the runs would alternate predictably (for the exact instructions, see the Supplemental Material available online).

We used a modified version of a task that was originally designed to explore the relationship between expectancy and response latencies under conditions of uncertainty (McAndrew, Yeates, Verbruggen, & McLaren, 2013).^1^ The trial sequence is shown in Figure 1b. Each trial started with the presentation of a blank screen for 3 to 4 s. Then, a brown cylinder (11 × 7 cm) was presented in the center of the screen against a white background, and subjects had to rate the extent to which they thought the no-go stimulus would appear, using a scale from 1 to 9 (1 = *definitely not a no-go trial*, 5 = *I do not know either way*, 9 = *definitely a no-go trial*). They gave this rating using their right hands on the numerical keypad of the computer keyboard. After 5 s, the words “peanut butter” or “brown sugar” appeared. For half of the subjects, “peanut butter” was the go stimulus and “brown sugar” was the no-go stimulus; for the other half, this mapping was reversed.

Subjects were instructed to respond as quickly as possible when the go stimulus appeared, but to withhold their response when the no-go stimulus appeared. Go responses were made with the left index finger, using a mouse. The mouse was mounted vertically with the buttons’ surfaces facing laterally; subjects pressed the mouse button by moving the left index finger inward in a lateral abduction. This movement is optimal for measuring electromyographic activity in the index finger (i.e., in the first dorsal interosseous muscle; for a similar setup with numerical keypads, see, e.g., Claffey, Sheldon, Stinear, Verbruggen, & Aron, 2010). The go stimulus remained on screen until a response was made, but the no-go stimulus disappeared after 2 s. If subjects responded to the no-go stimulus, an error sound was presented as feedback. Go and no-go stimuli were presented with equal probability. There were 208 trials in this experiment, split into eight blocks of 26 trials. We analyzed sequential effects, so we excluded the first trial of each block from the analyses. Subjects could move around between blocks. Whether the experiment started with a run of go trials or no-go trials was counterbalanced across subjects.

### Transcranial magnetic stimulation

Subjects completed a TMS safety-screening questionnaire and were found to be free of contraindications. After they had completed the questionnaire, they were seated in a chair with a mounted chin rest, which helped maintain head position. The left hand and inner forearm were cleaned with alcohol before attachment of the MEP electrodes. Two surface Ag/AgCl hydrogel electrodes (EL501; BIOPAC Systems, Goleta, CA) were positioned over the first dorsal interosseous muscle on the left hand, and a ground electrode was placed on the left inner forearm. A snug-fitting cap and earplugs were provided for the subject, and a subject tracker was positioned on the center of the forehead.

We used a MagStim 200-2 system with a BiStim module (Magstim, Whitland, Wales, United Kingdom) and a figure-8 coil (7-cm diameter) to deliver TMS pulses, and Brainsight software (Version 2.2.10; Rogue Research, Montréal, Quebec, Canada) to record MEPs and track the position of the coil. The TMS-calibration phase began with a few test pulses to ensure that the subject was comfortable. If the subject was happy to continue, we identified the optimal spot for eliciting MEPs in the left first dorsal interosseous muscle by looking for a visually perceptible movement that was isolated to the left index finger. This hot spot was marked as the target position relative to the subject tracker using the Brainsight software. At this point, the TMS coil was fixed over the motor hot spot, and the subject’s head was fixed using a head restraint and the chin rest. We then determined the resting motor threshold by finding the lowest stimulus intensity that produced MEPs of at least 50-µV amplitude on at least 5 of 10 trials (Rossini et al., 1994). Next, the subject’s 1,000-µV threshold was determined by finding the stimulus intensity that produced MEPs of approximately 1,000 µV at rest. Once the 1,000 µV threshold was found, the restraint was removed and the behavioral procedure was explained. During the experiment, the subject’s head was fixed again.

TMS pulses were delivered at one of two different time points in a trial, either 2.5 s into the blank interval (Pulse 1) or during the cylinder presentation after the prediction rating (Pulse 2). The delivery of Pulse 2 was contingent on the subjects making a rating, which could be done at any point during the 5-s presentation. If a prediction was not made (2.13% of the trials), a pulse was delivered at the end of this 5-s period. Of the 208 trials, 104 were Pulse 1 trials and 104 were Pulse 2 trials.

### Data analysis

All data files and R analysis scripts have been deposited in Open Research Exeter (http://hdl.handle.net/10871/19257).

#### Expectancy ratings

**Fig. 2.**
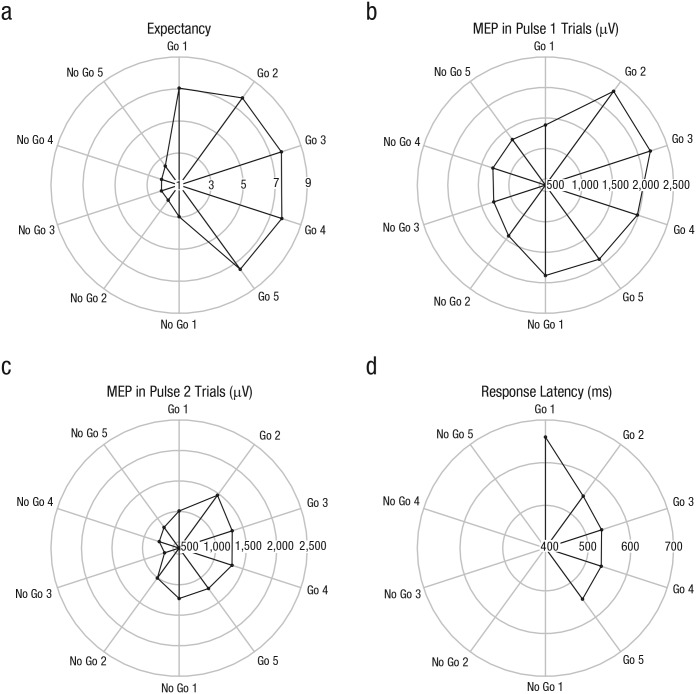
Radar plots of (a) expectancy ratings, the amplitude of motor evoked potentials (MEPs) on (b) Pulse 1 trials and (c) Pulse 2 trials, and (d) response latency on go trials. Different values of the dependent variables are represented by the concentric rings, and different trials are represented by the spokes. For expectancy ratings, 1 indicates that subjects strongly expected a no-go trial, and 9 indicates that they strongly expected a go trial. MEPs were recorded from the left first dorsal interosseous muscle after transcranial magnetic stimulation of the right primary motor cortex. Superimposing one graph on another allows comparison of different kinds of data.

To allow a direct comparison with the MEP data (Fig. 2), we converted raw expectancy scores (converted score = 10 – raw expectancy score) so that higher scores indicate that subjects expected a go trial (9 = *definitely a go trial*, 1 = *definitely a no-go trial*). Because we were particularly interested in the transitions from go trials to no-go trials and vice versa, we used paired *t* tests and the corresponding Bayes factors (for further details, see Table 1) to compare the expectancy ratings for the first trial of a go run (Go-1 trials; Fig. 1a) with the ratings for the adjacent last trial of a no-go run (No-Go-5 trials) and the second trial of a go run (Go-2 trials); similarly, we compared No-Go-1 trials (the first trial of a run of no-go trials) with Go-5 trials (the last trial of a go run) and No-Go-2 trials (the second trial of a no-go run). We calculated Bayes factors because they can provide support for the null hypothesis (i.e., there is no difference between trial types; Dienes, 2014).

**Table 1.** Overview of Paired *t* Tests Exploring the Effects of Run Transition on the Dependent Variables

Dependent variable and trial comparison	Difference between trials	95% CI	*t*(15)	*p*	*g*_av_	Bayes factor
Expectancy (1–9)						
No Go 5 – Go 1	−4.58	[−6.28, −2.88]	5.754	**.001**	2.600	670.410
Go 1 – Go 2	−0.67	[−1.37, 0.03]	2.039	.059	0.375	1.316
Go 5 – No Go 1	4.52	[2.97, 6.07]	6.220	**.001**	2.632	1,419.490
No Go 1 – No Go 2	0.82	[−0.15, 1.79]	1.793	.093	0.483	0.938
MEP in Pulse 1 trials (mV)						
No Go 5 – Go 1	−62	[−223, 99]	0.820	.425	0.060	0.343
Go 1 – Go 2	−915	[−1,371, −459]	4.277	**.001**	0.727	54.604
Go 5 – No Go 1	19	[−288, 326]	0.132	.897	0.014	0.257
No Go 1 – No Go 2	454	[−18, 925]	2.049	.058	0.337	1.334
MEP in Pulse 2 trials (mV)						
No Go 5 – Go 1	−188	[−380, 5]	2.08	.055	0.234	1.394
Go 1 – Go 2	−462	[−891, −32]	2.29	**.037**	0.393	1.905
Go 5 – No Go 1	−8	[−328, 313]	0.053	.959	0.007	0.256
No Go 1 – No Go 2	219	[30, 409]	2.472	**.026**	0.208	2.522
Reaction time (ms)						
Go 1 – Go 2	109	[36, 183]	3.173	**.006**	0.497	8.023

Note: Hedges’s average *g* (*g*_av_) is the reported effect-size measure, as recommended by Lakens (2013). Significant results are presented in boldface (*p* < .05). MEP = motor evoked potential. The Bayes factor is an odds ratio: It is the probability of the data under one hypothesis relative to that under another (Wetzels et al., 2011). H_0_ is the hypothesis that there is no difference between the trial types; H_A_ is the hypothesis that there is a difference between the trial types. Each evidence category is associated with a particular range of Bayes factor values: < 0.33 indicates substantial evidence for H_0_; 0.34–1 indicates anecdotal evidence for H_0_; 1 indicates no evidence; > 1–3 indicates anecdotal evidence for H_A_; > 3–10 indicates substantial evidence for H_A_; > 10 indicates strong to decisive evidence for H_A_. We calculated the Bayes factors reported in the table and the meta-analytic Bayes factors reported in the Results section with the BayesFactor package (Version 0.9.11-1; Morey, Rouder, & Jamil, 2015) for the R Software environment (Version 3.2.3.; R Development Core Team, 2015), using the default prior (i.e., 0.707). CI = confidence interval.

For completeness, we also report in the Appendix the results of a repeated measures analysis of variance (ANOVA) with run type (go or no-go) and run position (first, second, third, fourth, or fifth position in a run; see Fig. 1a) as within-subjects factors and expectancy as the dependent variable. Initially, a three-factor ANOVA was run on the expectancy ratings incorporating the variables pulse, run type, and run position to determine whether pulse influenced the expectancy data. There was no main effect of pulse (*p* = .57), and pulse did not interact with the run variables (all *p*s > .35), so the Pulse 1 and Pulse 2 data were collapsed.

#### MEPs

Peak-to-peak MEP amplitude was defined by the Brainsight software as the difference between the minimum and maximum electromyographic signal 10 to 90 ms after TMS delivery. Any trial on which the coil had drifted more than 7 mm away from the defined motor hot spot was excluded from the MEP analyses (6.75%). This exclusion criterion was determined in advance (on the basis of pilot work, which showed that MEP amplitude decreased substantially when the coil moved too far from the hot spot). We did not exclude any other trials. To examine pre-TMS baseline activity, we calculated root-mean-square electromyographic activity in the 50 ms preceding the TMS pulse for each condition, and we found it to be generally low (mean activity = 26 µV, *SD* = 51).

For both Pulse 1 and Pulse 2 trials, we used paired *t* tests and Bayes factors to compare the transitions from go trials to no-go trials and vice-versa (see Expectancy Ratings). Similar numerical patterns were observed for Pulse 1 and Pulse 2 trials (see Figure 2 and the difference scores in Table 1).^2^ Thus, to make optimal use of the information provided by the MEP measures, we calculated meta-analytic Bayes factors for multiple *t* tests (Rouder & Morey, 2011). Finally, we also ran two-way ANOVAs to assess the impact of run type and run position on MEPs.

#### Reaction times

Any individual reaction time (RT) that was more than 2 standard deviations away from the individual subject’s mean RT was excluded from analyses. The data were averaged and analyzed as a function of run position (see Expectancy Ratings). Note that RT data were produced only on go trials. Initially, a two-factor ANOVA was run on the RT data incorporating the variables pulse and run position to determine whether pulse influenced the RT data. There was no main effect of pulse (*p* = .27), and pulse did not interact with run position (*p* = .77), so the Pulse 1 and Pulse 2 data were collapsed.

#### Go and no-go accuracy

The percentages of missed go responses and incorrect responses on no-go trials (false alarms) appear in Table A1 in the appendix. Because percentages were very low, we did not analyze them further.

## Results

The expectancy ratings, MEPs, and RTs for go trials appear in Table A1 in the appendix and in Figure 2. Table 1 provides a detailed overview of the pairwise comparisons, and Table A2 in the appendix provides an overview of the corresponding univariate analyses. Note that the data pattern looked qualitatively similar when we included only go trials for which the expectancy rating was 8 or 9 (“definitely a go trial”) and no-go trials for which the rating was 1 or 2 (“definitely a no-go trial”; see the Supplemental Material).

Figure 2 shows that subjects kept track of the run sequence:^3^ The expectancy rating was significantly higher (i.e., subjects expected a go trial) for the first trial of a go run (Go 1; rating = 7.05) than for the last trial of a no-go run (No Go 5; rating = 2.47), *p* = .001, Bayes factor = 670.41 (Table 1), whereas it was significantly lower for the first trial of a no-go run (No Go 1; rating = 2.97) than for the last trial of a go run (Go 5; rating = 7.49), *p* = .001, Bayes factor = 1,419.49. Thus, subjects immediately adjusted their expectancy ratings when a new run started. The comparisons of Go-1 and Go-2 trials and of No-Go-1 and No-Go-2 trials (Table 1) suggest that some subjects further adjusted their expectancy ratings after the first trial of a run. However, these within-run differences (i.e., Go 1 – Go 2 and No Go 1 – No Go 2) were small compared with the between-run differences (i.e., No Go 5 – Go 1 and Go 5 – No Go 1; see Table 1).

Unlike the expectancy-rating pattern, the MEP pattern was not consistent with the run sequence (Fig. 2). As mentioned in the Method section, we calculated meta-analytic Bayes factors, combining the Pulse 1 and Pulse 2 *t* tests shown in Table 1. The meta-analyses revealed large within-run differences: Figure 2 shows that MEP increased substantially after the first go trial (meta-analytic Bayes factor = 242.66) and decreased substantially after the first no-go trial (meta-analytic Bayes factor = 12.10), both of which provide strong evidence for a difference between the trial types. Note that the univariate analysis also provided support for the idea that MEP changed within runs (see Table A2 in the appendix): The significant Run Type × Run Position interactions indicate that corticospinal excitability changed not only between runs (as shown by the main effects) but also within runs (as shown by the interactions). The between-run differences were numerically smaller than the within-run differences (see effect sizes in Table 1) or even absent: The No Go 5 – Go 1 comparison was inconclusive (meta-analytic Bayes factor = 1.11), but the Go 5 – No Go 1 comparison provided substantial evidence for the null hypothesis (i.e., there were no MEP difference between the trial types, meta-analytic Bayes factor = 0.19). Thus, the meta-analytic Bayes factors and inspection of the effect sizes (Table 1) indicate that the fluctuations in expectancy ratings and the MEP data did not correspond: The expectancy analyses revealed large between-run differences but small within-run differences; by contrast, the MEP analyses revealed little or no between-run differences but large within-run differences.

We observed a substantial RT difference between the first trial of a go run (Go 1; 660 ms) and the second trial of the go run (Go 2; 550 ms), *p* = .006, Bayes factor = 8.02 (Table 1), which is consistent with the MEP data. Thus, the expectancy data and the RT data are out of phase as well (Fig. 2): RT was longer after a run of no-go trials than after a single go trial, even though the runs were entirely predictable (and expectancy ratings indicated that subjects were fully aware of the run sequence).

## Discussion

We examined top-down control in a go/no-go task in which subjects knew in advance whether they had to execute a go response or not. We anticipated that the expectancy ratings, MEPs, and RTs would correspond in predictable environments because the top-down control system could bias actions. The expectancy ratings confirmed that subjects kept track of the run sequence: They anticipated a go trial at the beginning of a run of go trials and a no-go trial at the beginning of a run of no-go trials. To our surprise, the expectancy ratings were not reflected in the MEP and RT data. Motor excitability changed substantially after the first trial of a run, but it did not change much between runs. This finding suggests that motor excitability was associated primarily with the response properties of the previous trial (i.e., a no-go or go response), rather than the predicted properties of the current trial. The RT analysis also revealed a substantial cost at the beginning of a run: RTs were longer for Go-1 trials than for Go-2 trials, even though subjects clearly anticipated a go response on both trials.

How did previous events influence performance if the between-trial effects were not due to expectancy and top-down control? The MEP differences during the intertrial interval (i.e., for Pulse 1; see Table 1) indicate that the changes in corticospinal excitability and go latencies are not due solely to the retrieval of associations between the cue or stimulus and the go/no-go response (Perruchet et al., 2006). Instead, our results appear to be consistent with neural plasticity accounts (Dorris, Paré, & Munoz, 2000). Previous work suggests that residual activity of motor systems could contribute to sequential effects in action-control tasks (Kirby, 1976). Furthermore, single-cell studies indicate that the motor system is quickly altered by recent experiences, which produces longer-lasting effects; such automatic changes in the response bias of the action-control system could form the initial building blocks of motor learning (Dorris et al., 2000). More generally, our results are also consistent with hierarchical models of motor control in which the details of processing in the inner loop (motor preparation and corticospinal excitability) are not necessarily available to the outer loop (expectancy; Logan & Crump, 2011).^4^

Previous studies have shown that bottom-up influences modulate actions in unpredictable environments. The present study shows that such influences still modulate actions in entirely predictable environments. Models of cognitive control and goal-directed behavior propose that people create expectations about upcoming events and proactively modify the activity of perceptual and motor systems accordingly. Our results indicate that advance action control may be restricted in nature. Top-down control and proactively biasing response options are effortful (e.g., Braver, 2012), and most people prefer to avoid it when possible (Kool, McGuire, Rosen, & Botvinick, 2010). Thus, even though people can predict what will happen next, they may not always adjust their behavior accordingly. Such “failures to engage” (De Jong, 2000, p. 357) could open the door for the more bottom-up influences on action control we have identified in the current study. Future studies can explore whether motivating people and encouraging proactive control can reduce the bottom-up influences on MEPs and RTs in both predictable and unpredictable environments.

## Supplementary Material

Supplementary material

## Appendix

**Table A1.** Means and Standard Deviations for the Dependent Variables of Interest

Variable	Go 1	Go 2	Go 3	Go 4	Go 5	No Go 1	No Go 2	No Go 3	No Go 4	No Go 5
Expectancy (1–9)										
Mean	7.05	7.72	7.71	7.74	7.49	2.97	2.16	2.16	2.15	2.47
*SD*	1.62	1.87	1.99	1.98	1.93	1.41	1.88	1.95	1.95	1.82
MEP on Pulse 1 trials (µV)										
Mean	1,384	2,299	2,207	1,983	1,899	1,880	1,426	1,291	1,305	1,322
*SD*	975	1,481	1,773	1,392	1,252	1,442	1,181	972	1,184	1,052
MEP on Pulse 2 trials (µV)										
Mean	1,007	1,469	1,315	1,311	1,218	1,226	1,007	650	743	820
*SD*	827	1,465	1,274	1,120	1,076	1,169	885	588	625	734
Reaction time (ms)										
Mean	660	550	538	537	548	—	—	—	—	—
*SD*	275	154	159	162	175	—	—	—	—	—
Accuracy (% incorrect)										
Mean	0.0	0.0	0.0	0.0	0.0	4.7	0.6	0.0	0.2	0.4
*SD*	0.0	0.0	0.0	0.0	0.0	4.1	1.2	0.0	0.8	1.5

Note: MEP = motor evoked potential.

**Table A2.** Overview of Univariate Analyses Exploring the Effect of Run Type and Run Position on Expectancy Ratings, Motor Evoked Potentials (MEP), and Response Latencies

Variable	Sum of squares effect	Sum of squares error	*F*	*p*	Generalized η^2^
Expectancy					
Run type	1,065.49	493.08	*F*(1, 15) = 36.40	**.001**	.675
Run position	0.14	7.86	*F*(4, 60) = 0.26	.740	< .001
Run Type × Run Position	13.48	60.95	*F*(4, 60) = 3.31	.077	.025
MEP in Pulse 1 trials					
Run type	10,391,891	10,748,643	*F*(1, 15) = 14.50	**.002**	.039
Run position	1,428,150	10,542,930	*F*(4, 60) = 2.03	.142	.006
Run Type × Run Position	10,736,847	22,211,297	*F*(4, 60) = 7.25	**.003**	.041
MEP in Pulse 2 trials					
Run type	5,624,981	7,104,247	*F*(1, 15) = 11.88	**.004**	.035
Run position	1,351,374	9,365,766	*F*(4, 60) = 2.16	.137	.008
Run Type × Run Position	3,853,323	11,419,358	*F*(4, 60) = 5.06	**.014**	.024
RT					
Run position	175,327	268,273	*F*(4, 60) = 9.80	**.001**	.060

Note: Reaction time (RT) data were produced only on go trials. Greenhouse-Geisser corrected *p* values are reported. Significant results are presented in boldface (*p* < .05). MEP = motor evoked potential.

## Notes

1. Our experiment initially included a between-subjects condition in which the go/no-go sequence was unpredictable. We focus on the predictable condition only because there were not enough observations for the longer run lengths (run positions Go 4 or Go 5 and No Go 4 or No Go 5 in Fig. 1a) in the unpredictable condition as a result of an issue with the randomization. Furthermore, the effect of go and no-go runs is expected to be much smaller in the unpredictable condition, so much larger sample sizes would be needed to obtain sufficient statistical power in this condition.

2. MEP amplitude was generally lower for Pulse 2 than for Pulse 1. This could be due to the execution of the expectancy response with the other hand or to a general braking mechanism employed before response execution (Duque, Lew, Mazzocchio, Olivier, & Ivry, 2010).

3. At the end of the experiment, most subjects (*n* = 14) mentioned that they counted the number of trials to keep track of the sequence.

4. We thank our pal Gordon for suggesting this idea.
