# Supplementary material for: Limits of Executive Control: Sequential Effects in Predictable Environments
Source: Psychol Sci. 2016 Mar 21;27(5):748–57. doi: 10.1177/0956797616631990 (PMC4873728; doi:10.1177/0956797616631990)
Supplement: Supplementary material [file DS_10.1177_0956797616631990.pdf]

Supplementary Materials

Instructions for the Experiment

Half of the participants were given the following scenario: "*In this experiment you are a paramedic equipped to administer adrenaline. You are called out to see a number of people. Half have a nut allergy and half are diabetic. Each person has eaten a meal before calling you. The meal will be represented on screen as a brown cylinder. Sometimes the cylinder will represent peanut butter and sometimes brown sugar. You will see patients in rounds of 5, i.e. 5 nut allergy patients followed by 5 diabetic patients followed by 5 nut allergy patients and so on. Whenever you see a brown cylinder you are to rate the extent you think the patient is going to have eaten brown sugar and will need insulin. You do this using the numerical keypad with your right hand pressing one of nine buttons. They range from: 1 (I definitely think the patient will not need insulin), to 5 (I do not know either way) to 9 (I definitely think the patient will need insulin). If the patient has eaten peanut butter and needs adrenaline, press the bottom mouse key as fast as you can to administer the adrenaline. However, if the patient has eaten brown sugar you do not need to administer adrenaline so do not press anything to pass them on to another medic who will deal with them. You will hear an intermittent clicking throughout the experiment, this is the equipment taking measurements from you. Please ignore this. There will be 8 blocks of patients in this experiment in between which you should take a short break and start the next block when the experimenter is ready*". The go/no-go mapping was reversed for the other participants (i.e. brown sugar = go; peanut butter = no-go), so instructions were changed accordingly. Note that all participants had to rate the extent they thought the patient had eaten the substance that did not require a response (i.e. the no-go stimulus).

Additional results

We have plotted the averages after excluding go trials for which the expectancy rating was lower than 8, and no-go trials was higher than 2.

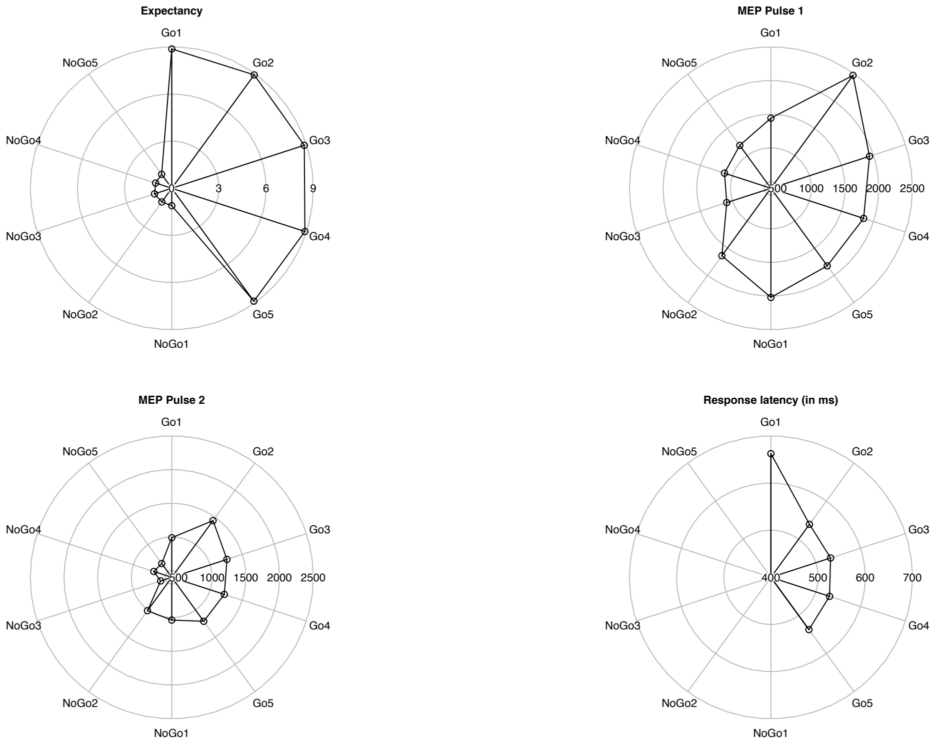

As can be seen, the results are very similar to the results reported in the main manuscript. A comparison with the expectancy panel shows that the MEP and expectancy data are misaligned: despite large differences in expectancy ratings, MEPs on Go1 trials are similar to the MEPs for NoGo5 trials; however, they increase substantially after the first go trial (i.e. Go2-Go5). Similarly, MEPs for NoGo1 trials are similar to the MEPs for Go5 trials, but they decreases throughout the nogo run. There was also a large RT cost at the beginning of a go run. In sum, the MEP and RT pattern is not consistent with the expectancy pattern.

We did not perform statistical tests because of missing observations for a few subjects (most of their expectancy ratings were between 3 and 7) and a relatively low number of observations for a few other subjects.
